# Use of a point-of-care test to rapidly assess levels of SARS-CoV-2 nasal neutralising antibodies in vaccinees and breakthrough infected individuals

**DOI:** 10.1038/s41598-023-47613-8

**Published:** 2023-11-20

**Authors:** Chee Wah Tan, Chuan Kok Lim, Jacqueline Prestedge, Mitchell Batty, Yun Yan Mah, Michelle O’Han, Lin-Fa Wang, Dean Kilby, Danielle E. Anderson

**Affiliations:** 1 Programme in Emerging Infectious Diseases, Duke-NUS Medical School, Singapore, 169857 Singapore; 2 Infectious Diseases Translational Research Programme, Department of Microbiology and Immunology, Yong Loo Lin School of Medicine, National University of Singapore, Singapore, 117547 Singapore; 3 Victorian Infectious Diseases Reference Laboratory, Melbourne Health, The Peter Doherty Institute for Infection and Immunity, Melbourne, 3000 Australia; 4 Department of Infectious Diseases, The Peter Doherty Institute for Infection and Immunity, The University of Melbourne, Melbourne, 3000 Australia; 5 Impact Biotech Healthcare, Level 30 Australia Square, 264 George St, Sydney, NSW 2000 Australia; 6 Department of Microbiology and Immunology, The Peter Doherty Institute for Infection and Immunity, The University of Melbourne, Melbourne, 3000 Australia

**Keywords:** Immunological techniques, SARS-CoV-2

## Abstract

Despite SARS-CoV-2 vaccines eliciting systemic neutralising antibodies (nAbs), breakthrough infections still regularly occur. Infection helps to generate mucosal immunity, possibly reducing disease transmission. Monitoring mucosal nAbs is predominantly restricted to lab-based assays, which have limited application to the public. In this multi-site study, we used lateral-flow surrogate neutralisation tests to measure mucosal and systemic nAbs in vaccinated and breakthrough infected individuals in Australia and Singapore. Using three lateral flow assays to detect SARS-CoV-2 nAbs, we demonstrated that nasal mucosal nAbs were present in 71.4 (95% CI 56.3–82.9%) to 85.7% (95% CI 71.8–93.7%) of individuals with breakthrough infection (positivity rate was dependent upon the type of test), whereas only 20.7 (95% CI 17.1–49.4%) to 34.5% (95% CI 19.8–52.7%) of vaccinated individuals without breakthrough infection had detectible nasal mucosal nAbs. Of the individuals with breakthrough infection, collective mucosal anti-S antibody detection in confirmatory assays was 92.9% (95% CI 80.3–98.2%) of samples, while 72.4% (95% CI 54.1–85.5%) of the vaccinated individuals who had not experienced a breakthrough infection were positive to anti-S antibody. All breakthrough infected individuals produced systemic anti-N antibodies; however, these antibodies were not detected in the nasal cavity. Mucosal immunity is likely to play a role in limiting the transmission of SARS-CoV-2 and lateral flow neutralisation tests provide a rapid readout of mucosal nAbs at the point-of-care.

## Introduction

Since 2019, Severe Acute Respiratory Syndrome Coronavirus 2 (SARS-CoV-2) has gradually evolved through the emergence of variants with a predisposition for upper respiratory tropism, higher transmissibility and lower severity^1,2^. Systemic mRNA vaccines for SARS-CoV-2 have been shown to be highly effective in reducing disease severity and hospitalisation. In May 2023, the WHO declared an end to the public health emergency^3^, signalling a change in how the pandemic is managed and monitored moving forward.

Modelling has shown a strong correlation between viral neutralising antibody titres with protection against symptomatic infection with SARS-CoV-2 variants^4^. This information is useful in predicting the level of protective immunity and decay in immunity over time post-vaccination or infection^5^. The effect on transmission is less prominent, possibly due to lower levels of protective mucosal antibodies in relation to the sera. Nasal humoral immunity has been increasingly recognised as an important element in preventing SARS-CoV-2 transmission or limiting the infection to the upper respiratory tract^6,7^. Most of the evidence derives from animal challenge studies, in which intranasal vaccines or monoclonal antibodies protect against infection with SARS-CoV-2^6^. In addition, low levels of anti-SARS-CoV-2 antibodies are often detected in nasal secretions following systemic vaccinations, despite much higher antibody levels in the sera. It is possible that the emergence of more immune-evasive variants (such as various Omicron subvariants) in combination with low nasal immunity induced by systemic vaccination could contribute to increased susceptibility to infection^8^.

Immunity is traditionally assessed using virus neutralisation tests (VNTs) but the main challenges with this method are the lack of run-to-run and inter-laboratory consistencies and the low throughput nature of such in vitro assays. The development of surrogate virus neutralisation tests (sVNTs) based on antibody-mediated blockage of ACE2-spike protein interaction has allowed for scalable population surveillance, with strong correlation to VNT when compared to other conventional assays targeting spike receptor binding domains (S-RBD)^9^. Although the sVNT assay was developed to assess anti-SARS-CoV-2 neutralising antibody levels in blood or serum, these scalable assays are also an attractive tool in assessing nasal humoral immunity at a population level for surveillance and disease modelling purposes. It is imperative to understand the mucosal neutralising antibody response to SARS-CoV-2 (especially in the nasal cavity) elicited by infection or from vaccination, and sVNT assays have been used in an attempt to do so^10^. However, the sVNT assay is not practical outside laboratory settings.

Lateral flow assays (LFAs) provide an accessible clinical utility that is not limited by the need for expensive equipment or a well-equipped laboratory. Thus, we undertook a proof-of-concept study using three independently manufactured SARS-CoV-2 neutralising antibody detection LFAs to determine whether these types of assays could be used to reliably detect and measure neutralising antibodies (nAbs) from the nasal mucosa. Following on, a small-scale longitudinal observational study was conducted to assess the nasal immunity of laboratory workers in two laboratories in Australia and Singapore. We found that LFAs were able to detect nAbs in nasal secretions, and this was particularly evident following infection.

As COVID-19 surveillance programmes evolve in many countries towards more community focussed testing using rapid point-of-care tests, innovative approaches such as the development of sVNT technologies in LFA format are highly appealing for rapid, large-scale population surveillance, especially when accessing remote and vulnerable populations.

## Methods

### Study participants

Human experimental work was conducted according to the Declaration of Helsinki Principles and the Australian National Health and Medical Research Council Code of Practice. All participants provided verbal informed consent prior to the study. Ethical approval for this project was obtained from the Royal Melbourne Hospital Human Research Ethics Committee (RMH HREC 2020.179) and Duke-NUS Medical School (NUS-IRB-2021-108).

A total of 71 volunteers were included in this study (Table 1). 51 volunteers were recruited in Australia and 20 in Singapore. All participants had received at least 3 COVID-19 vaccine doses at the time of participation. Participants were required to fill out a questionnaire to indicate the date and manufacturer of each vaccine they had received, and the dates of any previous SARS-CoV-2 infections (confirmed by PCR or rapid antigen test). New SARS-CoV-2 infections that arose during the study period were also noted, with a second set of samples collected from those participants (Table 2).

**Table 1:** Participant demographics.

Demographics			SARS-CoV-2 vaccination history									SARS-CoV-2 infection history			Sample collection			Longitudinal
Participant ID	Gender	Age (years)	Vaccinated	Dose 1		Dose 2		Dose 3		Dose 4		Previous Infection	Infection 1	Infection 2	Date of blood/swab sample collection	Time since most recent infection (days)	Time since last vaccination (days)	Date of post-study PCR or RAT
				Date	Vaccine	Date	Vaccine	Date	Vaccine	Date	Vaccine		Date of first PCR or RAT	Date of first PCR or RAT				
NLFA-001	F	45	Yes	1/21/2021	Pfizer	2/11/2021	Pfizer	11/6/2021	Pfizer			Yes	12/05/2022		10/26/2022	167	354	
NLFA-002	F	26	Yes	20/05/2021	Pfizer	10/06/2021	Pfizer	23/12/2021	Pfizer			Yes	26/06/2022		10/27/2022	123	308	
NLFA-003	F	27	Yes	12/04/2021	Pfizer	06/05/2021	Pfizer	25/02/2022	Pfizer			Yes	17/01/2022	13/10/2022	10/26/2022	13	243	
NLFA-004	M	42	Yes	18/03/2021	Pfizer	08/04/2021	Pfizer	12/11/2021	Pfizer	09/08/2022	Pfizer	No			10/26/2022	–	78	12/11/2022
NLFA-005	F	39	Yes	01/09/2021	Pfizer	04/10/2021	Pfizer	08/01/2022	Pfizer	11/07/2022	Moderna	No			10/26/2022	–	107	11/29/2022
NLFA-007	M	31	Yes	3/19/2021	Pfizer	4/19/2021	Pfizer	11/14/2021	Pfizer			Yes	3/29/2022		10/28/2022	213	348	12/10/2022
NLFA-008	M	59	Yes	08/04/2021	Pfizer	08/04/2021	Pfizer	14/12/2021	Pfizer			Yes	30/05/2022		10/28/2022	151	318	
NLFA-009	F	29	Yes	22/05/2021	Pfizer	13/06/2021	Pfizer	16/12/2021	Pfizer			Yes	27/03/2022		10/28/2022	215	316	
NLFA-010	M	46	Yes	19/03/2021	Pfizer	13/04/2021	Pfizer	19/11/2021	Pfizer	29/07/2022	Moderna	No			10/28/2022	–	91	
NLFA-011	M	54	Yes	31/05/2021	AstraZeneca	29/08/2021	AstraZeneca	14/03/2022	Pfizer			Yes	19/10/2022		10/28/2022	9	228	
NLFA-012	F	61	Yes	25/06/2021	Pfizer	16/07/2021	Pfizer	17/12/2021	Pfizer	19/07/2022	Pfizer	No			11/3/2022	–	107	11/20/2022
NLFA-013	F	37	Yes	19/03/2021	Pfizer	12/04/2021	Pfizer	08/11/2021	Pfizer	12/08/2022	Novavax	No			11/3/2022	–	83	
NLFA-014	F	52	Yes	28/05/2021	Pfizer	28/06/2021	Pfizer	21/12/2021	Pfizer	16/07/2022	Pfizer	Yes	1/9/2021		11/3/2022	663	110	
NLFA-015	F	43	Yes	27/09/2021	Pfizer	18/10/2021	Pfizer	07/02/2022	AstraZeneca			No			11/3/2022	–	269	
NLFA-016	F	53	Yes	06/04/2021	AstraZeneca	24/06/2021	AstraZeneca	10/12/2021	Pfizer	21/07/2022	Pfizer	No			11/3/2022	–	105	
NLFA-017	F	36	Yes	19/08/2021	AstraZeneca	14/10/2021	AstraZeneca	09/03/2022	Moderna			No			11/3/2022	–	239	11/25/2022
NLFA-018	M	60	Yes	4/6/2021	AstraZeneca	6/29/2021	AstraZeneca	12/15/2021	Pfizer	8/23/2022	Pfizer	Yes	5/15/2022		11/7/2022	176	76	
NLFA-019	F	50	Yes	08/07/2021	Pfizer	29/07/2021	Pfizer	20/12/2021	Moderna	7/11/2022	Moderna	No			11/7/2022	–	119	
NLFA-020	M	65	Yes	7/15/2021	Pfizer	8/6/2021	Pfizer	1/5/2022	Pfizer	7/19/2022	Pfizer	No			11/7/2022	–	111	
NLFA-021	M	33	Yes	06/06/2021	Pfizer	02/07/2021	Pfizer	22/12/2021	Moderna			Yes	27/06/2022		11/7/2022	133	320	
NLFA-022	F	55	Yes	12/04/2021	AstraZeneca	12/07/2021	AstraZeneca	21/12/2021	Pfizer	12/04/2022	Pfizer	No			11/7/2022	–	209	
NLFA-023	M	31	Yes	09/03/2021	Pfizer	30/03/2021	Pfizer	29/10/2021	Pfizer			Yes	20/04/2022		11/7/2022	201	374	
NLFA-024	F	49	Yes	6/2/2021	Pfizer	7/16/2021	Pfizer	12/23/2021	Pfizer			Yes	5/25/2022		11/23/2022	182	335	
NLFA-025	M	33	Yes	5/13/2021	Pfizer	6/5/2021	Pfizer	12/22/2021	Pfizer	8/23/2022	Pfizer	Yes	6/1/2022		11/23/2022	175	92	
NLFA-026	F	53	Yes	4/9/2021	AstraZeneca	7/2/2021	AstraZeneca	7/14/2021	Pfizer	7/28/2022	Pfizer	Yes	1/9/2022		11/28/2022	323	123	
NLFA-027	F	58	Yes	4/6/2021	AstraZeneca	6/29/2021	AstraZeneca	12/17/2021	Pfizer			Yes	6/28/2022		11/28/2022	153	346	
NLFA-028	F	55	Yes	6/3/2021	Pfizer	6/29/2021	Pfizer	12/16/2021	Pfizer	7/20/2022	Novavax	Yes	4/18/2022		11/28/2022	224	131	
NLFA-029	M	43	Yes	12/03/2021	Pfizer	06/04/2021	Pfizer	19/11/2021	Pfizer			Yes	30/06/2022		11/28/2022	151	374	
NLFA-030	M	45	Yes	3/18/2021	Pfizer	4/19/2021	Pfizer	11/8/2021	Pfizer			Yes	7/28/2022		11/28/2022	123	385	
NLFA-031	M	67	Yes	3/21/2021	Pfizer	4/6/2021	Pfizer	12/17/2021	Pfizer	5/11/2022	Moderna	No			11/28/2022	–	201	12/23/2022
NLFA-032	F	59	Yes	3/18/2021	Pfizer	4/12/2021	Pfizer	11/17/2021	Pfizer			No			11/28/2022	–	376	
NLFA-033	M	44	Yes	3/18/2021	Pfizer	4/18/2021	Pfizer	11/15/2021	Pfizer			Yes	7/28/2022		11/28/2022	123	378	
NLFA-034	F	67	Yes	3/18/2021	Pfizer	4/15/2021	Pfizer	11/18/2021	Pfizer			Yes	1/19/2022		11/28/2022	313	375	
NLFA-035	F	64	Yes	3/21/2021	Pfizer	4/16/2021	Pfizer	12/17/2021	Pfizer	5/23/2022	Moderna	No			11/28/2022	–	189	12/25/2022
NLFA-036	F	54	Yes	6/3/2021	AstraZeneca	8/30/2021	AstraZeneca	1/16/2022	Moderna	7/24/2022	Moderna	No			12/15/2022	–	144	
NLFA-037	F	61	Yes	25/06/2021	Pfizer	16/07/2021	Pfizer	17/12/2021	Pfizer	19/07/2022	Pfizer	Yes	11/20/2022		12/15/2022	25	149	
NLFA-038	M	45	Yes	3/20/2020	Pfizer	4/9/2021	Pfizer	12/17/2021	Pfizer			Yes	04/02/2022	08/08/2022	12/15/2022	129	363	
NLFA-039	F	39	Yes	01/09/2021	Pfizer	04/10/2021	Pfizer	08/01/2022	Pfizer	11/07/2022	Moderna	Yes	11/29/2022		12/15/2022	16	157	
NLFA-040	M	46	Yes	4/6/2021	AstraZeneca	7/6/2021	AstraZeneca	1/6/2022	Pfizer			No			12/15/2022	–	343	
NLFA-041	M	31	Yes	4/23/2021	Pfizer	5/15/2021	Pfizer	1/10/2022	Pfizer			Yes	5/27/2022		12/15/2022	202	339	
NLFA-042	F	43	Yes	4/27/2021	Pfizer	5/20/2021	Pfizer	12/1/2021	Pfizer			Yes	11/28/2022		12/15/2022	17	379	
NLFA-043	F	36	Yes	19/08/2021	AstraZeneca	14/10/2021	AstraZeneca	09/03/2022	Moderna			Yes	11/25/2022		12/15/2022	20	281	
NLFA-044	F	57	Yes	6/13/2021	Pfizer	7/5/2021	Pfizer	1/21/2022	Moderna			No			12/15/2022	–	328	
NLFA-045	F	43	Yes	7/12/2021	Pfizer	8/9/2021	Pfizer	1/6/2022	Pfizer	7/19/2022	Moderna	No			12/15/2022	–	149	
NLFA-046	M	28	Yes	3/16/2021	Pfizer	4/16/2021	Pfizer	11/8/2021	Pfizer			No			12/21/2022	–	408	12/24/2022
NLFA-047	F	59	Yes	3/15/2021	Pfizer	4/6/2021	Pfizer	11/8/2021	Pfizer	11/14/2022	Moderna bivalent	Yes	6/9/2022		12/21/2022	195	37	
NLFA-048	M	31	Yes	3/19/2021	Pfizer	4/19/2021	Pfizer	11/14/2021	Pfizer			Yes	3/29/2022	12/10/2022	12/21/2022	11	402	
NLFA-049	M	49	Yes	3/15/2021	Pfizer	4/18/2021	Pfizer	12/19/2021	Pfizer	8/11/2022	Pfizer	Yes	4/19/2022		1/11/2023	267	153	
NLFA-050	M	67	Yes	3/21/2021	Pfizer	4/6/2021	Pfizer	12/17/2021	Pfizer	5/11/2022	Moderna	Yes	12/23/2022		1/11/2023	19	245	
NLFA-051	F	64	Yes	3/21/2021	Pfizer	4/16/2021	Pfizer	12/17/2021	Pfizer	23/05/2022	Moderna	Yes	12/25/2022		1/11/2023	17	233	
NLFA-052	M	28	Yes	3/16/2021	Pfizer	4/16/2021	Pfizer	11/8/2021	Pfizer			Yes	12/24/2022		12/21/2022	–	408	
NLFA-053	F	39	Yes	1/23/2021	Pfizer	2/15/2021	Pfizer	11/26/2021	Moderna			Yes	6/28/2022		12/15/2022	170	384	
NLFA-054	F	28	Yes	1/18/2021	Pfizer	2/8/2021	Pfizer	11/22/2021	Moderna	16/12/2022	Moderna bivalent	No			11/28/2022	–	371	
NLFA-055	M	31	Yes	1/18/2021	Pfizer	2/8/2021	Pfizer	11/16/2021	Moderna			No			11/28/2022	–	377	12/22/2022
NLFA-056	M	33	Yes	1/18/2021	Pfizer	2/8/2021	Pfizer	1/25/2022	Moderna			No			11/28/2022	–	307	
NLFA-057	M	37	Yes	1/18/2021	Pfizer	2/8/2021	Pfizer	10/15/2021	Pfizer			No			11/28/2022	–	409	
NLFA-058	F	32	Yes	6/3/2021	Moderna	7/15/2021	Moderna	3/14/2022	Pfizer	19/11/2022	Moderna bivalent	No			11/28/2022	–	9	
NLFA-059	M	35	Yes	1/23/2021	Pfizer	2/14/2021	Pfizer	11/21/2021	Moderna			Yes	8/8/2022		11/28/2022	112	372	
NLFA-060	F	32	Yes	1/23/2021	Pfizer	2/14/2021	Pfizer	12/28/2021	Moderna			No			11/30/2022	–	337	
NLFA-061	F	34	Yes	1/23/2021	Pfizer	2/14/2021	Pfizer	11/28/2021	Pfizer			Yes	7/1/2022		11/28/2022	150	365	
NLFA-062	M	47	Yes	1/22/2021	Pfizer	2/14/2021	Pfizer	10/10/2021	Moderna			No			11/29/2022	–	415	
NLFA-063	M	34	Yes	1/23/2021	Pfizer	2/14/2021	Pfizer	12/3/2021	Pfizer	9/12/2022	Moderna bivalent	Yes	8/14/2022		11/30/2022	108	362	
NLFA-064	M	34	Yes	1/23/2021	Pfizer	2/14/2021	Pfizer	1/4/2021	Pfizer			Yes	10/4/2022		11/29/2022	56	694	
NLFA-065	M	30	Yes	1/22/2021	Pfizer	2/14/2021	Pfizer	10/16/2021	Moderna			No			11/29/2022	–	409	
NLFA-066	M	36	Yes	1/22/2021	Pfizer	2/14/2021	Pfizer	10/13/2021	Moderna	11/11/2022	Moderna bivalent	Yes	8/4/2022		11/29/2022	117	18	
NLFA-067	M	35	Yes	6/4/2021	Moderna	7/16/2021	Moderna	3/18/2022	Pfizer			Yes	8/6/2022		11/28/2022	114	255	
NLFA-068	F	32	Yes	6/6/2021	Moderna	7/18/2021	Moderna	3/11/2022	Pfizer			No			11/29/2022	–	263	
NLFA-069	F	26	Yes	6/13/2021	Moderna	7/17/2021	Moderna	12/23/2021	Moderna			Yes	9/13/2022		11/28/2022	76	340	
NLFA-070	F	27	Yes	6/2/2021	AstraZeneca	8/4/2021	Moderna	12/28/2021				Yes	10/12/2022		11/28/2022	47	335	
NLFA-071	F	32	Yes	7/5/2021	Pfizer	8/7/2021	Pfizer	12/31/2021	Moderna			No			11/28/2022	–	332	
NLFA-072	F	25	Yes	6/21/2021	Pfizer	7/23/2021	Pfizer	1/4/2022	Moderna	15/12/2022	Moderna	Yes	2/10/2022		11/28/2022	291	328

**Table 2:** Reinfection cohort.

Demographics		SARS-CoV-2 vaccination history								SARS-CoV-2 infection history			Sample collection		
Participant ID	Age (years)	Dose 1		Dose 2		Dose 3		Dose 4		Previous infection	Infection 1	Infection 2	Date of blood/swab sample collection	Days since most recent infection	Days since last vaccination
		Date	Vaccine	Date	Vaccine	Date	Vaccine	Date	Vaccine		Date of first PCR or RAT	Date of first PCR or RAT			
NLFA-005	39	01/09/2021	Pfizer	04/10/2021	Pfizer	08/01/2022	Pfizer	11/07/2022	Moderna	No			10/26/2022	–	107
NLFA-039										Yes	11/29/2022		12/15/2022	16	157
NLFA-012	61	25/06/2021	Pfizer	16/07/2021	Pfizer	17/12/2021	Pfizer	19/07/2022	Pfizer	No			11/3/2022	–	107
NLFA-037										Yes	11/20/2022		12/15/2022	25	149
NLFA-017	36	19/08/2021	AstraZeneca	14/10/2021	AstraZeneca	09/03/2022	Moderna			No			11/3/2022	–	237
NLFA-043										Yes	11/25/2022		12/15/2022	20	279
NLFA-007	31	3/19/2021	Pfizer	4/19/2021	Pfizer	11/14/2021	Pfizer			Yes	3/29/2022		10/28/2022	–	348
NLFA-048										Yes	3/29/2022	12/10/2022	12/21/2022	11	402
NLFA-031	67	3/21/2021	Pfizer	4/6/2021	Pfizer	12/17/2021	Pfizer	5/11/2022	Moderna	No			11/28/2022	–	201
NLFA-050										Yes	12/23/2022		1/11/2023	19	245
NLFA-035	64	3/21/2021	Pfizer	4/16/2021	Pfizer	12/17/2021	Pfizer	5/23/2022	Moderna	No			11/28/2022	–	189
NLFA-051										Yes	12/25/2022		1/11/2023	17	216
NLFA-046	28	3/16/2021	Pfizer	4/16/2021	Pfizer	11/8/2021	Pfizer			No			12/21/2022	–	308
NLFA-052										Yes	12/24/2022		1/11/2023	18	329

### Sample collection

All human specimen materials were considered infectious and hazardous and handled using standard biosafety procedures. Serum: Samples of blood were obtained by venepuncture, collected in red top Vacutainer^®^ collection tubes without the presence of coagulants. Blood was allowed to clot, and the serum separated by centrifugation at 3000 × g for 10 min. The serum was then carefully withdrawn and decanted into a new pre-labelled tube. Specimens were frozen at − 20 °C for longer term storage and tested as soon as possible after thawing. For frozen samples, more than two freeze–thaw cycles were avoided. Prior to testing, frozen specimens were brought to room temperature slowly and gently mixed. Samples containing visible particulate matter were clarified by centrifugation before testing. Whole blood: Samples of blood were obtained by venepuncture, collected in purple top Vacutainer^®^ collection tubes containing heparin. Heparinised blood was collected and used for LFA within 3 h of collection. Nasal swabs: Each individual participant performed their own nasal swab (both nostrils were swabbed with a nylon flocked applicator) and swab was immersed in 1 mL liquid Amies media and used for LFA within 3 h of collection. Remaining nasal lysate sample was stored at − 20 °C and subsequently used for immunoassays.

### Qualitative colloidal gold lateral flow assays

Two LFAs (SARS-CoV-2 Neutralising Antibody Rapid Test Kit, DXK007, GenScript; and COVID-19 IgM/IgG Antibody Colloidal Gold Test, Anbio) were used for each sample type. The test sample (50 µL whole blood or 50 µL nasal lysate) was dispensed into the sample well of the test cassette. The sample mixes with the colloidal gold RBD conjugates upon sample addition and anti-SARS-CoV-2 RBD antibodies, if present, bind to the RBD-biotin-Au conjugates. Samples were allowed to migrate for 15 min, then LFA cassettes were photographed. Two visible lines on the cassette indicate a SARS-CoV-2 RBD antibody positive test result.

### Quantitative fluorescent lateral flow assay

The Neutralising Antibody of SARS-CoV-2 Test (Fluorescence Immunochromatographic Assay) (Anbio) was used for each sample type. The test sample (50 µL whole blood or 50 µL nasal lysate) was dispensed into the sample well of the test cassette. Samples were allowed to migrate for 15 min, then LFA cassettes were read in the handheld immunofluorescence analyser (AF-100S, Anbio) and values recorded. A value above 25.0 IU/mL indicates a SARS-CoV-2 RBD antibody positive test result.

### Electro-chemiluminescence immunoassay (ECLIA)

Two assays were used: the Elecsys® Anti-SARS-CoV-2 assay (Roche), which uses a recombinant nucleocapsid (N) protein to detect N antibodies; and the Elecsys® Anti-SARS-CoV-2 S assay (Roche), which uses a recombinant RBD protein to detect Spike antibodies. Testing was undertaken on the Roche Cobas platform, as per the manufacturer’s instructions for use. For the Elecsys® Anti-SARS-CoV-2 assay, semi-quantitative results are reported as a cut-off index (COI), where a COI < 1.0 is non-reactive and a COI ≥ 1.0 is reactive. For the Elecsys® Anti-SARS-CoV-2 S assay, the analyser calculates the analyte concentration of each sample in U/mL, where < 0.80 U/mL is negative, ≥ 0.80 to ≤ 250 U/mL is positive and ≥ 250 U/mL is positive and the numeric value is reported as ≥ 250 U/mL.

### Multiplex immunoassay

SARS-CoV-2 N and S protein conjugated MagPlex microspheres (Luminex) were pre-incubated with 1:100 diluted serum or nasal swab sample for 1 h at 37 °C, followed by addition of 1:1000 diluted PE-labelled, anti-human IgG antibody (eBioscience) for 1 h at 37 °C. The MFI value was acquired using MAGPIX luminex platform. Samples with MFI value greater than 800 were considered positive.

### Ethics statement

Ethical approval for this project was obtained from the Royal Melbourne Hospital Human Research Ethics Committee (RMH HREC 2020.179) and Duke-NUS Medical School (NUS-IRB-2021-108).

## Results

### Participant demographics and samples

**Figure 1:**
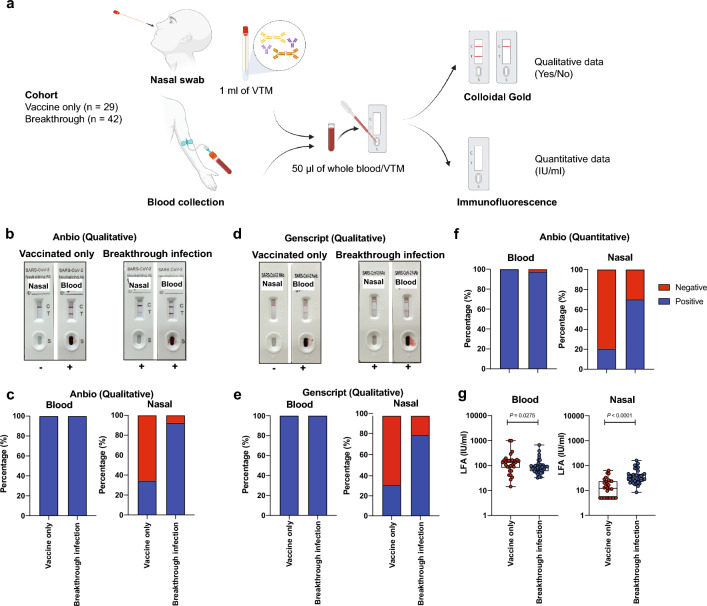
(**a**) Sample collection and procedure. A total of 71 participants were recruited in Australia and Singapore. A nasal swab and a venous blood sample were collected from each participant. Three lateral flow assays were simultaneously used to test the two samples from each participant. (**b**, **c**) Anbio, and (**d**, **e**) GenScript qualitative colloidal gold neutralisation test. (**f**, **g**) Anbio quantitative fluorescence neutralisation test.

To establish if nAbs could be detected by Point-of-Care (POC) lateral flow devices, we sampled 71 participants from two countries: Australia (AU) and Singapore (SG) (Fig. 1a). Of the 71 participants, 51 were in Australia and 20 in Singapore. There were 39 females and 32 males in the cohort with a median age of 42 (range 25–67). All participants had received at least 3 COVID-19 vaccine doses, predominantly Pfizer and Moderna, but also AstraZeneca and Novavax. Only vaccinated participants were recruited into this study (Table 1).

### Detection of neutralising antibodies by LFA

We tested each sample, simultaneously, on three lateral flow assays: two qualitative colloidal gold LFAs (Anbio and Genscript) and one quantitative immunofluorescence LFA (Anbio) (Fig. 1a). Both qualitative LFAs positively detected nAbs from the blood samples of all participants, as expected. Nasal nAbs were detected in 60.6% (95% CI 48.9–71.1%) of samples by Genscript LFA and 64.8% (95% CI 53.2–74.9%) by Anbio (Fig. 1b–d). Nasal nAbs were predominantly detected in individuals with breakthrough infection, but the rate of positivity varied between LFA. In the vaccinated only cohort, positivity rates of 34.5% (95% CI 19.8–52.7%) (Anbio, Fig. 1c) and 31.0% (17.1–49.4%) (Genscript, Fig. 1e), were observed in qualitative assays and 20.7% (95% CI 9.5–38.7%) in the quantitative assay (Anbio, Fig. 1f). In the breakthrough infection cohort, positivity rates of 80.9% (95% CI 66.4–90.3%), 85.7% (95% CI 71.8–93.7%), and 71.4% (95% CI 56.3–82.9%) were detected (Fig. 1c, e, f respectively). In summary, all three LFAs were able to detect SARS-CoV-2 nAbs in the nasal samples of vaccinated individuals or breakthrough-infected individuals.

We observed that the proportion of individuals with nasal nAbs was higher in the breakthrough infection group of the cohort (Fig. 1g). When comparing the geometric mean of the level of circulating nAbs in the blood, detected by LFA, we found vaccinated individuals had higher levels (119 IU/mL), compared with individuals with breakthrough infection (88 IU/mL) (Fig. 1g). In contrast, significantly higher levels of nasal nAbs (35.9 IU/mL) were detected in individuals with breakthrough infections compared with uninfected participants (11.9 IU/mL) (Fig. 1g). These results suggest that nasal mucosal immunity is enhanced following an infection.

### Serum vs nasal antibodies

**Figure 2:**
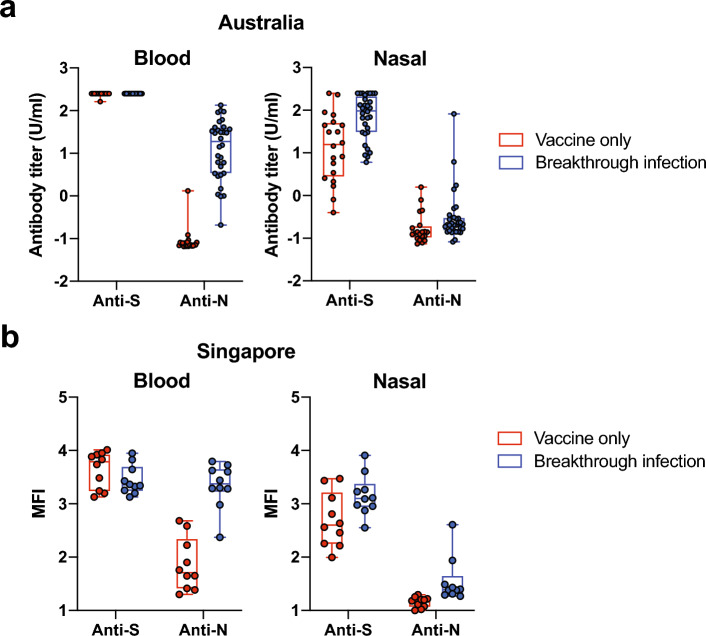
Nasal and blood spike and N antibody levels detected in blood and nasal swab from (**a**) Australia and (**b**) Singapore. In Australia, the N and Spike antibodies were detected using Elecsys^®^ Anti-SARS-CoV-2 assay (Roche), which uses a recombinant nucleocapsid (N) protein to detect N antibodies; and the Elecsys^®^ Anti-SARS-CoV-2 S assay (Roche), which uses a recombinant RBD protein to detect Spike antibodies, while a Luminex-based microsphere assay was used in Singapore. Each dot denotes the antibody titre (U/ml or MFI) of a sample, while the box shows the interquartile range with median at the centre, and the whiskers represent the maximum and minimum.

To further characterise blood and nasal samples from both cohorts (AU and SG), and to differentiate between spike (S) and nucleocapsid (N) antibodies, two distinct assays were used. The ECLIA assay was used to analyse the AU cohort (Fig. 2a) and multiplex microsphere assay was used for the SG cohort (Fig. 2b). S antibodies were detected in all participants using either assay. S antibodies were readily detectable in nasal samples, but present in a higher percentage of people following breakthrough infection (72.4% and 95.4% in vaccinated and breakthrough infection, respectively). All individuals (100%) who had prior exposure to SARS-CoV-2 carried N antibodies in the blood. N antibodies were not readily detected in nasal samples; only 9.3% (95% CI 3.1–22.2%) of the infected individuals had detectable N antibodies. Infection increased the amount of S, but not N, antibodies in the nasal cavity, however the majority of the readings that arose from the N assay were relatively low and below the limit of detection.

### Agreement between assays

**Figure 3:**
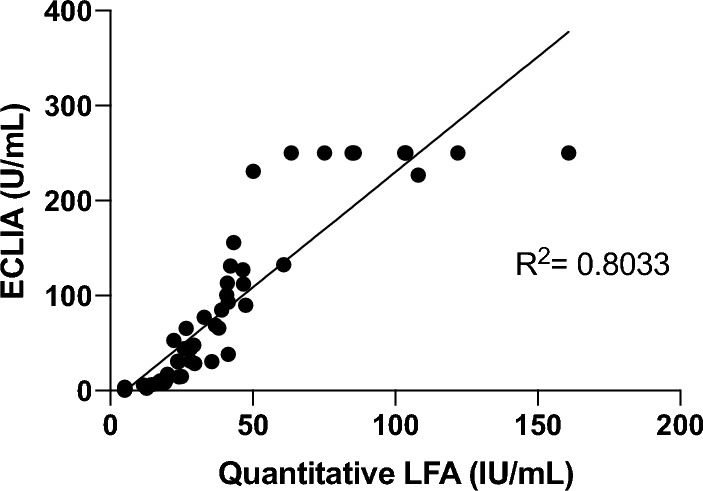
Correlation of neutralising antibodies across assays. Values obtained from the AU cohort on the quantitative LFA were compared with a diagnostic electro-chemiluminescence immunoassay (ECLIA), Elecsys^®^ Anti-SARS-CoV-2 S. Spearman correlation analysis using the IU/ml value determined by quantitative LFA and U/mL value determined by ECLIA produces a correlation between two assays, with an R^2^ = 0.8033.

To determine specificity of the quantitative LFA to detect nAbs from nasal samples, we compared the results obtained on the LFA with a diagnostic electro-chemiluminescence immunoassay (ECLIA), Elecsys^®^ Anti-SARS-CoV-2 S, which uses a recombinant RBD protein to detect Spike antibodies (Fig. 3). The ECLIA assay is used as a diagnostic assay by the VIDRL lab, where the AU samples were collected, and the samples used in this comparison were from the AU cohort. Linear regression using the IU/ml value determined by quantitative LFA and U/mL value determined by ECLIA suggests a moderate correlation between two assays, with an R^2^ = 0.8033, and supported by an overall concordance between assays of 62.7% (95% CI 49–74.7%) and a Kappa statistic of 0.06 (95% CI − 0.05 to 0.2).

### Longevity of antibodies in blood and nasal secretions

**Figure 4:**
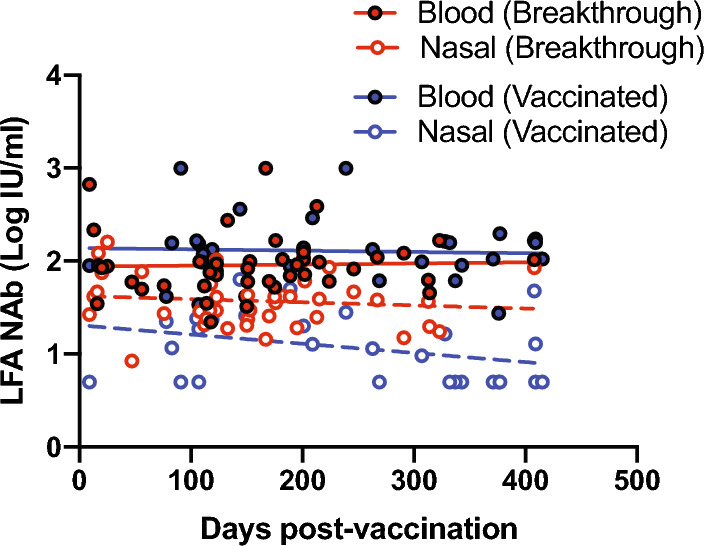
Longevity of nasal and blood nAbs in vaccinated or breakthrough-infected individuals. Each dot denotes the neutralising antibody titre (IU/ml) of an individual sample. Linear regression analysis was performed using GraphPad Prism 8.

We examined the longevity of blood and nasal nAbs as detected by quantitative LFA. We observed that neither vaccinated individuals, nor those who experienced a breakthrough infection showed waning of circulating blood nAbs, up until 15 months post-vaccination (Fig. 4). Compared with circulating antibodies in the blood, the overall level of nasal nAbs was quantitatively lower, and the antibodies in vaccinated individuals appeared to wane at a faster rate (Fig. 4). These results indicate that breakthrough infection produces higher levels of nAbs in the nasal mucosa and those antibodies are more robust than the antibodies found in nasal mucosa of those who had not experienced a breakthrough infection.

### Nasal antibodies increase after infection

**Figure 5:**
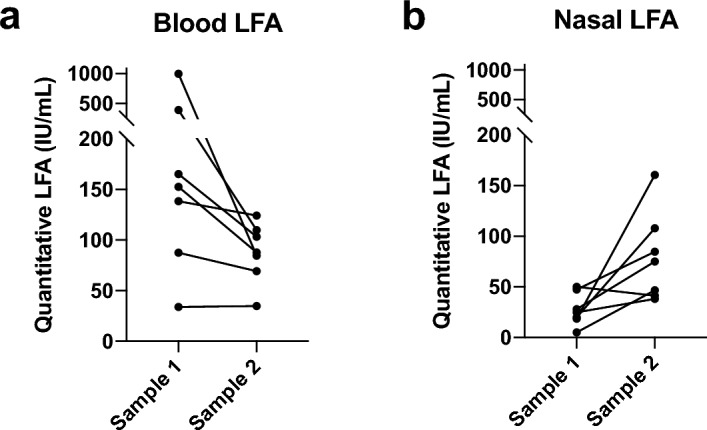
Neutralising antibody response pre- and post-breakthrough infection in (**a**) blood and (**b**) nasal samples.

During the course of the study, there were 7 participants in the AU cohort who contracted SARS-CoV-2 and a second sample set was collected following their infection (Table 2). Of these 7 participants, 6 were previously uninfected, and one had been infected once before. NAbs in blood and serum were measured by quantitative LFA. The level of nAbs in the blood decreased after infection in all but one participant (Fig. 5a). In contrast, detectable nasal nAbs increased in all but one participant (Fig. 5b). This data demonstrates that nasal nAbs increase to detectable levels after infection and can be measured by rapid test.

## Discussion

Nasal and/or mucosal immunity plays a critical role in the defence against airborne viral infection. Nasal IgA levels wane and are not induced by subsequent booster vaccinations^11^ and increasing numbers of SARS-CoV-2 breakthrough infections among vaccinated individuals indicate that long lived sterilising immunity is not maintained. This is likely a complex result of continued virus evolution to evade immune detection in the context of immunisation against ancestral WT and/or Omicron BA.1 antigen. Studies have shown that vaccines that elicit a robust mucosal immune response are more effective at preventing infection and transmission of SARS-CoV-2^12^. Therefore, elicited nasal or mucosal immunity is crucial for the effectiveness of COVID-19 vaccines. Nasally administered vaccines are under development that will, hopefully, induce sustained neutralising antibody production against SARS-CoV-2 at the site of viral entry^13–16^.

Currently, assessing mucosal nAbs remains primarily restricted to lab-based serological tests. In this study, we demonstrated that three separate POC lateral flow SARS-CoV-2 neutralisation tests were able to detect nAbs in the nasal cavity. Even though the POC assays used in the experiments were originally developed to measure nAbs in blood or serum, the extended functionality to measure nasal nAbs from a simple swab enables rapid and easy identification of adaptive protection against SARS-CoV-2 at the physiological site where it is most important to provide sterilising immunity. Such POC assays would allow for easier, increased surveillance testing of, for example, healthcare workers and staff members at skilled nursing facilities, which would potentially reduce COVID-19 cases and deaths among residents as previously shown by McGarry et al.^17^. As this is a preliminary study using these POC assays in a manner that differs from the manufacturer’s instructions, validation would be required before implementing their use in clinical settings.

This study shows that intramuscular administration of the original vaccines most commonly used in Australia and Singapore can elicit an immune response (in a proportion of the population) in the form of anti-S and neutralising antibody presence in the nasal cavities. The levels of both neutralising and total anti-S antibodies are significantly increased in people with breakthrough infections, with a concurrent decrease in serum nAbs. In addition, there was a moderate correlation between neutralising antibody and anti-S antibody levels. It is unclear at this stage as to whether the nasal mucosa nAbs are produced in situ or whether nAbs present in the serum translocate to the site of infection to defend against viral entry and replication.

Using different assays to measure the anti-S and anti-N antibodies across AU and SG cohorts was not ideal, however due to equipment availability in the laboratories this was a necessity. Even so, the results from both countries corresponded with anti-S and anti-N antibodies being detected in the blood of all participants who were vaccinated or had a breakthrough infection, respectively.

The persistent levels of nAbs that we found in both the blood and nasal mucosa is contradictory to the majority of reports that have measured nAbs from vaccinated subjects in longitudinal studies and shown a decrease over time^18–21^. Even though the wane rate differs between studies, it is generally accepted that it occurs (thus, the need for booster vaccinations) and the differences are reported to be due to different vaccine regimes, age, sex and/or comorbidities^22–24^. The method by which we detected nAbs is quite new and is potentially the reason for the unexpected results, although it is also accepted that POCT LFA are inherently less sensitive than traditional assays. Alternatively, the neutralising antibody response elicited among individuals differs greatly and measurement of the levels at a single point in time, as opposed to longitudinally, does not provide the most accurate wane rate indication. Irrespective of the reason, Levin et al.^22^ have previously shown a negligible decline in nAbs between 3 and 6 months post-vaccination and Tuells et al.^25^ similarly demonstrated the maintenance of optimal neutralising antibody levels 6 months after vaccination using a rapid lateral flow immunochromatography test. These reports support our results of sustained neutralising antibody levels.

This study has shown that both blood and nasal nAbs can be detected by qualitative and quantitative POC assays in a rapid and easy manner with only relatively small sample volumes required. Even with the ongoing research into the kinetics and dynamics of nAbs and how this may relate to the protection against SARS-CoV-2 infection, the relationship is still poorly understood and highly complex. A longitudinal study with a larger sample size will be conducted to measure nasal mucosa nAbs at regular intervals over a period of 12 months; such data will provide a more accurate indication of the longevity of the neutralising capacity, and associated protection against SARS-CoV-2, at the viral site of entry.

A few limitations with this study can be noted. As the participants for the study were laboratory workers who had all been vaccinated, there were no true negative control samples in the form of unvaccinated subjects. Furthermore, it is difficult to recruit unvaccinated or uninfected individuals as majority of the population are either vaccinated or infected. In addition, the sample size was not large enough to group by time post-vaccination or infection to more robustly assess the wane rate of nAbs in the blood or nasal mucosa in this manner. Furthermore, the nasal swabs were collected into 1 mL liquid Amies, with only 50 µL added to each device; this dilution factor is quite large relative to the blood samples, which were applied neat. Lastly, this study does not specifically measure mucosal IgA level and further analysis of the nAb isotypes is warranted as it has been shown IgG and IgA antibody concentrations provide different binding specificity, which is location, infection, time and vaccination type specific^26–28^.

Irrespective of the limitations, clear differences can be seen in neutralising, anti-S and anti-N antibody levels between subjects that have had breakthrough infections and those who have been vaccinated only. Similar results were seen by Liu et al.^10^ during their longitudinal study; the ‘hybrid’ immune model (infection followed by mRNA vaccine) showed greater induction of nAbs against ancestral SARS-CoV-2 and BA.1 variant in the nasal mucosa of participants who had been infected relative to those who had been vaccinated only.

In conclusion, we demonstrated that POCT LFA could detect nAbs from the nasal cavity, allowing surveillance of humoral mucosal immunity at the population level.

## Data Availability

All data are presented in the manuscript.

## References

[1] Balint G, Voros-Horvath B, Szechenyi A (2022). Omicron: Increased transmissibility and decreased pathogenicity. Signal Transduct. Target Ther..

[2] Halfmann PJ (2022). SARS-CoV-2 Omicron virus causes attenuated disease in mice and hamsters. Nature.

[3] Statement on the fifteenth meeting of the IHR (2005) Emergency Committee on the COVID-19 pandemic. https://www.who.int/news/item/05-05-2023-statement-on-the-fifteenth-meeting-of-the-international-health-regulations-(2005)-emergency-committee-regarding-the-coronavirus-disease-(covid-19)-pandemic#:~:text=The%20WHO%20Director%2DGeneral%20concurs,of%20international%20concern%20(PHEIC) (2022).

[4] Khoury DS (2021). Neutralizing antibody levels are highly predictive of immune protection from symptomatic SARS-CoV-2 infection. Nat. Med..

[5] Cromer D (2022). Neutralising antibody titres as predictors of protection against SARS-CoV-2 variants and the impact of boosting: A meta-analysis. Lancet Microbe.

[6] Mao T (2022). Unadjuvanted intranasal spike vaccine elicits protective mucosal immunity against sarbecoviruses. Science.

[7] Lin Y (2023). Nasal spray of neutralizing monoclonal antibody 35B5 confers potential prophylaxis against severe acute respiratory syndrome coronavirus 2 variants of concern: A small-scale clinical trial. Clin. Infect. Dis..

[8] Marking U (2023). 7-month duration of SARS-CoV-2 mucosal immunoglobulin-A responses and protection. Lancet Infect. Dis..

[9] Tan CW (2020). A SARS-CoV-2 surrogate virus neutralization test based on antibody-mediated blockage of ACE2-spike protein-protein interaction. Nat. Biotechnol..

[10] Liu S (2023). Comparison of the mucosal and systemic antibody responses in Covid-19 recovered patients with one dose of mRNA vaccine and unexposed subjects with three doses of mRNA vaccines. Front. Immunol..

[11] Liew F (2023). SARS-CoV-2-specific nasal IgA wanes 9 months after hospitalisation with COVID-19 and is not induced by subsequent vaccination. EBioMedicine.

[12] Christensen D (2022). Protection against SARS-CoV-2 transmission by a parenteral prime-Intranasal boost vaccine strategy. EBioMedicine.

[13] Wang Q (2023). Intranasal booster using an Omicron vaccine confers broad mucosal and systemic immunity against SARS-CoV-2 variants. Signal Transduct. Target Ther..

[14] Lam JY (2022). A nasal omicron vaccine booster elicits potent neutralizing antibody response against emerging SARS-CoV-2 variants. Emerg. Microbes Infect..

[15] Xiao L (2023). Mucosal SARS-CoV-2 nanoparticle vaccine based on mucosal adjuvants and its immune effectiveness by intranasal administration. ACS Appl. Mater. Interfaces.

[16] Sonvico F (2023). Nasal delivery as a strategy for the prevention and treatment of COVID-19. Expert. Opin. Drug. Deliv..

[17] McGarry BE, Gandhi AD, Barnett ML (2023). Covid-19 surveillance testing and resident outcomes in nursing homes. N. Engl. J. Med..

[18] Bayart JL (2021). Waning of IgG, total and neutralizing antibodies 6 months post-vaccination with BNT162b2 in healthcare workers. Vaccines (Basel).

[19] Barbeau DJ (2022). Comparative analysis of human immune responses following SARS-CoV-2 vaccination with BNT162b2, mRNA-1273, or Ad26.COV2.S. NPJ Vaccines.

[20] Chia WN (2021). Dynamics of SARS-CoV-2 neutralising antibody responses and duration of immunity: A longitudinal study. Lancet Microbe.

[21] Evans JP (2022). Neutralizing antibody responses elicited by SARS-CoV-2 mRNA vaccination wane over time and are boosted by breakthrough infection. Sci. Transl. Med..

[22] Levin EG (2021). Waning immune humoral response to BNT162b2 Covid-19 vaccine over 6 months. N. Engl. J. Med..

[23] Kolaric B, Ambriovic-Ristov A, Tabain I, Vilibic-Cavlek T (2021). Waning immunity six months after BioNTech/Pfizer COVID-19 vaccination among nursing home residents in Zagreb, Croatia. Croat. Med. J..

[24] Zhang R (2022). Immunogenicity of a heterologous prime-boost COVID-19 vaccination with mRNA and inactivated virus vaccines compared with homologous vaccination strategy against SARS-CoV-2 variants. Vaccines (Basel).

[25] Tuells J (2022). Detection of neutralizing antibodies against SARS-CoV-2 post-vaccination in health care workers of a large tertiary hospital in spain by using a rapid test LFIC and sVNT-ELISA. Vaccines (Basel).

[26] Froberg J (2023). Primary exposure to SARS-CoV-2 via infection or vaccination determines mucosal antibody-dependent ACE2 binding inhibition. J. Infect. Dis..

[27] Cao KT (2023). SARS-CoV-2 mRNA vaccination induces an intranasal mucosal response characterized by neutralizing antibodies. J. Allergy Clin. Immunol. Glob..

[28] Ishizaka A (2023). Research article antibody induction and immune response in nasal cavity by third dose of SARS-CoV-2 mRNA vaccination. Virol. J..

